# A Scholarly Knowledge Graph-Powered Dashboard: Implementation and User Evaluation

**DOI:** 10.3389/frma.2022.934930

**Published:** 2022-07-19

**Authors:** Olga Lezhnina, Gábor Kismihók, Manuel Prinz, Markus Stocker, Sören Auer

**Affiliations:** ^1^Learning and Skill Analytics Research Group, TIB–Leibniz Information Centre for Science and Technology, Hannover, Germany; ^2^Data Science and Digital Libraries Group, TIB–Leibniz Information Centre for Science and Technology, Hannover, Germany; ^3^Knowledge Infrastructures Research Group, TIB–Leibniz Information Centre for Science and Technology, Hannover, Germany; ^4^L3S Research Center, Leibniz University of Hannover, Hannover, Germany; ^5^TIB–Leibniz Information Centre for Science and Technology, Hannover, Germany

**Keywords:** dashboard, scholarly knowledge graph, ORKG, user evaluation, scholarly communication

## Abstract

Scholarly knowledge graphs provide researchers with a novel modality of information retrieval, and their wider use in academia is beneficial for the digitalization of published works and the development of scholarly communication. To increase the acceptance of scholarly knowledge graphs, we present a dashboard, which visualizes the research contributions on an educational science topic in the frame of the Open Research Knowledge Graph (ORKG). As dashboards are created at the intersection of computer science, graphic design, and human-technology interaction, we used these three perspectives to develop a multi-relational visualization tool aimed at improving the user experience. According to preliminary results of the user evaluation survey, the dashboard was perceived as more appealing than the baseline ORKG-powered interface. Our findings can be used for the development of scholarly knowledge graph-powered dashboards in different domains, thus facilitating acceptance of these novel instruments by research communities and increasing versatility in scholarly communication.

## Introduction

Knowledge graphs, as effective tools of information retrieval (Reinanda et al., 2020), are applied in various domains (Zou, 2020; Abu-Salih, 2021), including physics (Say et al., 2020), healthcare (Steenwinckel et al., 2020; Zhang et al., 2020), business (Meier et al., 2021), and education (Chen et al., 2018; Chi et al., 2018; Rizun, 2019; Qin et al., 2020). Scholarly knowledge graphs operate with academic literature (Turki et al., 2021) and thus can be viewed in the frame of the scholarly knowledge ecosystem (Altman and Cohen, 2022). Implementation of such novel technologies in scholarly communication is beneficial for Open Science (Ignat et al., 2021). The Open Research Knowledge Graph (ORKG) is a scholarly knowledge graph that implements the research contribution model (Vogt et al., 2020) encompassing actual results (contributions) of academic literature. The ORKG research service infrastructure initiative (https://www.orkg.org/orkg/about/1/Overview) integrates crowdsourcing and automated techniques for generating scholarly knowledge graphs (Jaradeh et al., 2019) which enable the user to compare research contributions (Oelen et al., 2019) and create FAIR (findable, accessible, interoperable, and reusable) literature surveys (Oelen et al., 2020). Scholarly knowledge graphs represent a cutting-edge technology that might be useful for resolving problems of contemporary scholarly communication, which lead to the replication crisis, such as limited findability of research; unequal access to published papers; deterioration of peer review quality; compromised research integrity; insufficient machine readability of literature; and restricted availability of open research tools (Guédon et al., 2019).

For the wider application of this relatively novel technology, it is necessary to make it more appealing to research communities in various academic areas (Sabou et al., 2018; Auer et al., 2020). Therefore, user-friendly interfaces for knowledge graphs are continuously developed (Portenoy et al., 2017; Vargas et al., 2019; Ortiz Vivar et al., 2020; Kurteva and De Ribaupierre, 2021), which take into account mechanisms of human information processing (Plumbley and Abdallah, 2006). Previous research stressed the importance of visual interfaces which employ principles of computer science, graphic design, and human-technology interaction (Cavaller, 2021). In accordance with information theory (Cole et al., 2002), visualizations can be helpful for schematizing relationships and aiding pattern recognition, thus optimizing the cognitive load of the user (Sweller et al., 2019). It was shown that reduced cognitive load (Hu et al., 2017; Sweller et al., 2019; Castro-Alonso et al., 2021) is vital for information processing, and ease-of-use is a crucial factor influencing human interaction with technology (Hassenzahl et al., 2010; Venkatesh et al., 2016; Lah et al., 2020).

For information visualization, dashboards are among the instruments of choice (Pauwels et al., 2009). A dashboard can be defined as a visual display of the most important information needed to achieve one or more objectives, consolidated on a single screen (Few, 2004). It is an interactive tool with dynamically updated data that allows information monitoring (Hayward, 2022). Although a scholarly knowledge graph-powered dashboard, to the best knowledge of the authors, has not been described in the literature, dashboards are increasingly used in different areas of research and practice: in medicine (Faiola et al., 2015), epidemiology (Center for Systems Science Engineering, 2021), or in the assessment of scientific conferences (Angioni et al., 2020).

In this article, we present a scholarly knowledge graph-powered dashboard developed as a user-friendly interface at the intersection of computer science, graphic design, and human-technology interaction. The code for running the service locally is in open access at https://github.com/OlgaLezhnina/dashboard. We also conducted a user evaluation survey to assess the results of our work. The survey data and the code are at https://github.com/OlgaLezhnina/dashboard_survey.

## Methods

### Open Research Knowledge Graph Dashboard

For the development of the ORKG dashboard, we considered the list of tasks that a scholarly knowledge graph should enable a researcher to complete: get a research field overview; find related work; assess relevance; extract relevant information; get recommended articles; obtain deep understanding; and reproduce results (Brack et al., 2021).

An interface with this functionality, the ORKG resource comparison (Oelen et al., 2019), was already implemented in the frame of the ORKG research service infrastructure initiative, and our task was to create an alternative version with the aim of increasing versatility in scholarly knowledge graph-powered interfaces. The resource comparison interface allows selecting research contributions, mapping their properties, and publishing the result online in a tabular form. Evaluation of user performance (Oelen et al., 2019) showed that the participants found the service useful and fairly intuitive. When working on new interface development, we realized that we could not venture to outmatch the existing one in terms of usefulness and performance. We sought improvement opportunities in the areas of graphic design and human-technology interaction, which was feasible considering the fact that the advantage of a dashboard is the visual presentation of information.

The requirements for the visual interface were elaborated by Cavaller (2021): multi-relational dynamic visualizations, such as dashboards, should aim for consistency in the selection of their content; schematicity in the formal representation of information; versatility in encoding and setup of visualization; appealingness in graphic design; accessibility of media channel; and effectiveness perceived by the user. Thus, we focused on the appeal and ease-of-use properties of the dashboard from the perspective of the user experience. These are interrelated characteristics, as it was shown that optimal ease-of-use is required for finding an informational input appealing and, specifically, interesting: excessively complicated or oversimplified presentation leads to decreased interest and reduced attention, thus stirring boredom (Tam et al., 2021).

**Figure 1:**
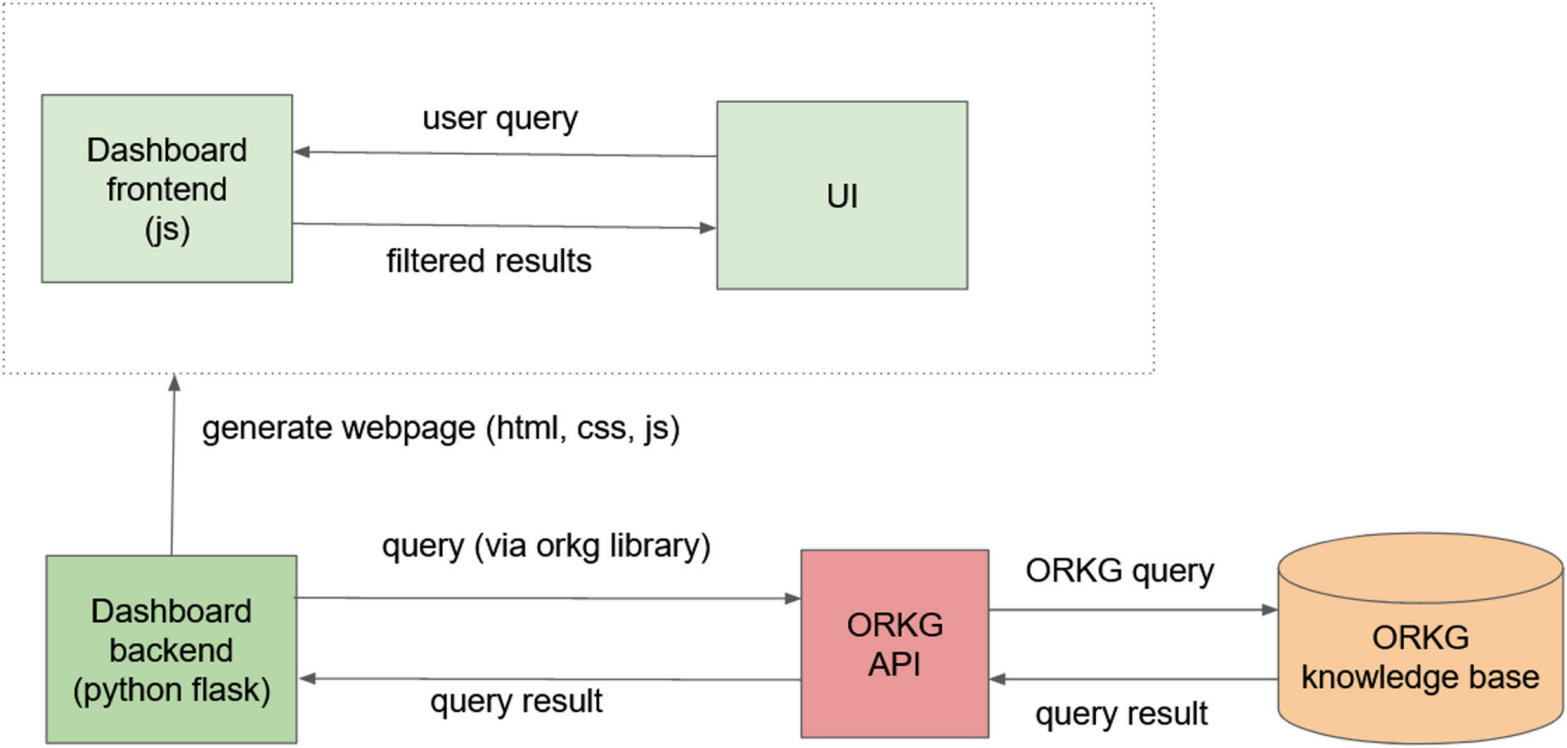
System architecture. ORKG, Open Research Knowledge Graph; UI, user interface; API, application programming interface.

The dashboard was implemented in accordance with these principles. The system architecture is shown in Figure 1. The backend is a Flask server (Python), with the orkg library used for queries and the pygal library used for the visual presentation of results. To generate a webpage, the backend queries the ORKG server to get all information required for the scope of the dashboard; the results are embedded into the generated webpage using Jinja templates. Any other operations are handled by the JavaScript frontend. When the users interact with the dashboard interface, the information stored in the webpage is queried and displayed to them. The presentation is dynamic, and when a new paper on the topic is added to the ORKG, the dashboard contents are automatically updated.

In terms of content, we chose a topic from educational science, attitudes toward information and communication technology (ICT) in the Programme for International Student Assessment (PISA) 2015 and 2018. We did not have specific requirements for the topic and selected a subject that two of the authors were researching at that moment and were therefore acquainted with relevant literature. An additional argument in favor of this topic was that the potential participants of the user evaluation survey would not be familiar with it and thus keep focused on evaluating the interface as such, without being influenced by their attitude to the topic. We selected relevant papers on the topic and added them to the ORKG knowledge base.

**Figure 2:**
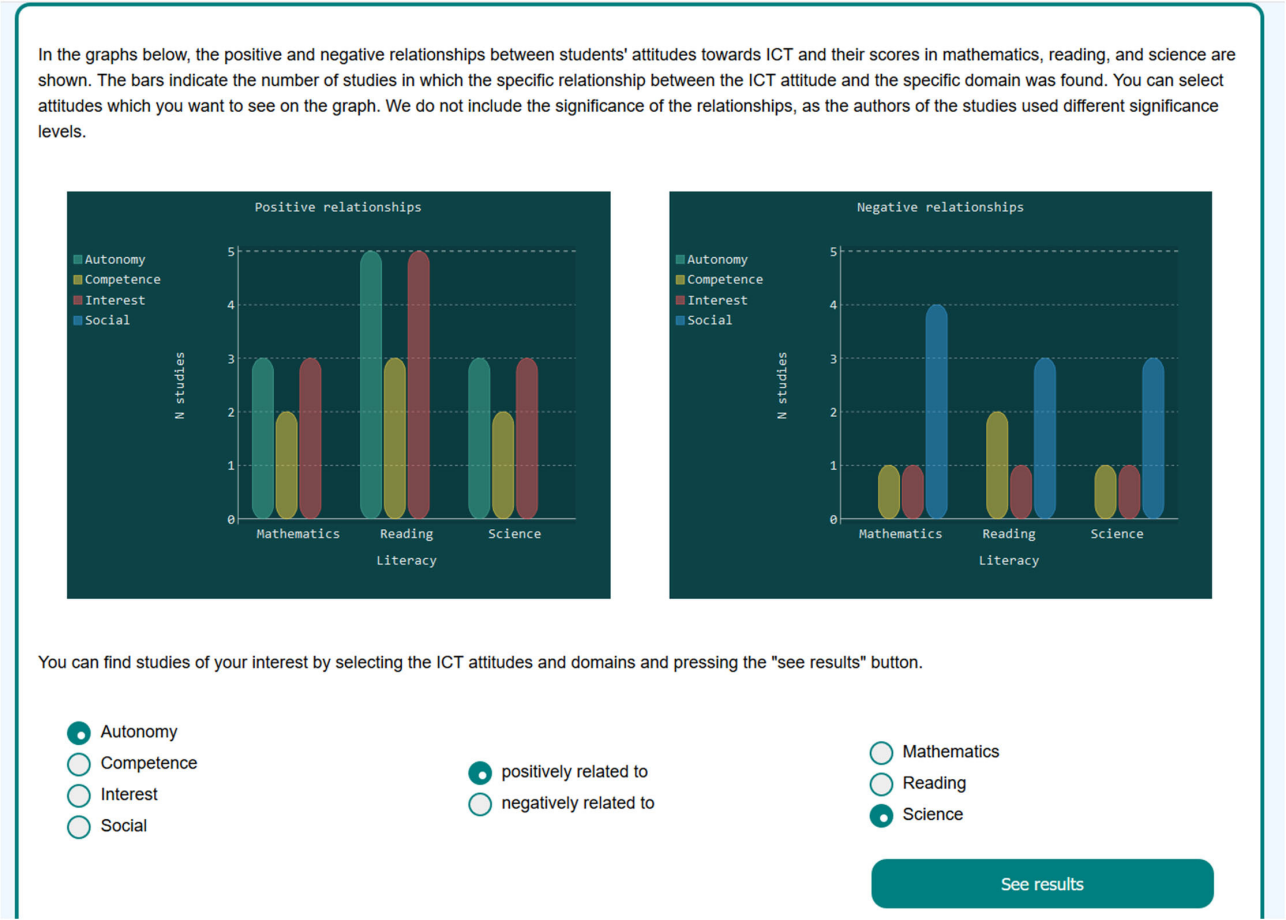
The dashboard barplots and radio buttons for selecting papers.

The properties of research contributions included datasets, participant countries, methodology, attitudes to ICT, outcome variables, and results (relationships between the attitudes and the outcome variables). The layout was designed to map the textual modality to the visual modality (Manovich, 2011). For visualizations, we chose countries and the results of the studies. For presenting countries, the geographic map of the world was plotted with the pygal library (Python). The user can hover over the map to see the number of studies referring to a specific country and use the dropdown menu for selecting studies based on the countries they include. In addition, the results of studies were visualized with the barplots (Figure 2) showing the number of studies with a specific finding, that is, a specific relationship between an attitude toward ICT and students' scores in mathematics, reading, or science. The users could select studies of their interest with the radio buttons below the plots.

The dashboard was implemented as a use case in the frame of the ORKG research service infrastructure initiative https://www.orkg.org/orkg/usecases/pisa-dashboard/. For additional information, we referred the user to the resource comparison on the topic https://www.orkg.org/orkg/comparison/R76906 and stressed that both interfaces are integral parts of the ORKG; they are not mutually exclusive but can complement each other. Our goal was to facilitate versatility in scholarly communication by widening the spectrum of knowledge graph-based interfaces. Therefore, we conducted an evaluation survey to figure out whether the new service can be a beneficial addition to the existing ORKG functionality. The resource comparison was used as a baseline for the evaluation of the ORKG dashboard, as we explain in detail in the next section.

### User Evaluation Survey

We invited participants *via* social media in professional groups interested in ORKG and Open Science. The participants were asked (1) to evaluate their experiences with the actual services (the ORKG dashboard and the relevant resource comparison); and (2) to assess the potential usefulness of similar services if implemented in their area of research. In Section A (the first task), we used the short version of the User Experience Questionnaire (the UEQ-S). The instrument was psychometrically validated (Schrepp et al., 2017; Hinderks et al., 2018). It consists of the eight pairs of opposite characteristics (confusing/clear, inefficient/efficient, complicated/easy, obstructive/supportive, boring/exciting, not interesting/interesting, conventional/inventive, and usual/leading edge), which the participant evaluates on the scale from −3 to +3. The UEQ-S questions were obligatory to answer. In Section B (the second task), we asked the participants to evaluate on the scale from 1 to 5, how advantageous similar services could be for different aspects of scholarly communication if implemented in their area of research. We listed five such aspects: get acquainted with a new topic; answer a specific question; get an overview of relevant research; explore novel methods of scholarly communication; and make their own research visible to others. The participants also evaluated (on the scale from 1 to 5) the overall usefulness of the dashboard and the resource comparison if jointly implemented in their area of research. Both parts of the survey included open questions so that the participants could comment on their experience with the dashboard and with the resource comparison separately and reflect on the idea of implementing both services in their area of research. Finally, as the science domain might influence the researchers' relation to scholarly communication (see Bu et al., 2021; Yan et al., 2021), we asked participants to give relevant information about themselves: whether they work in technical or humanitarian professions; conduct mostly quantitative or mostly qualitative research; and deal with academic literature rather frequently or only occasionally. Option “other” was included in each of these questions.

## Evaluation Results

**Figure 3:**
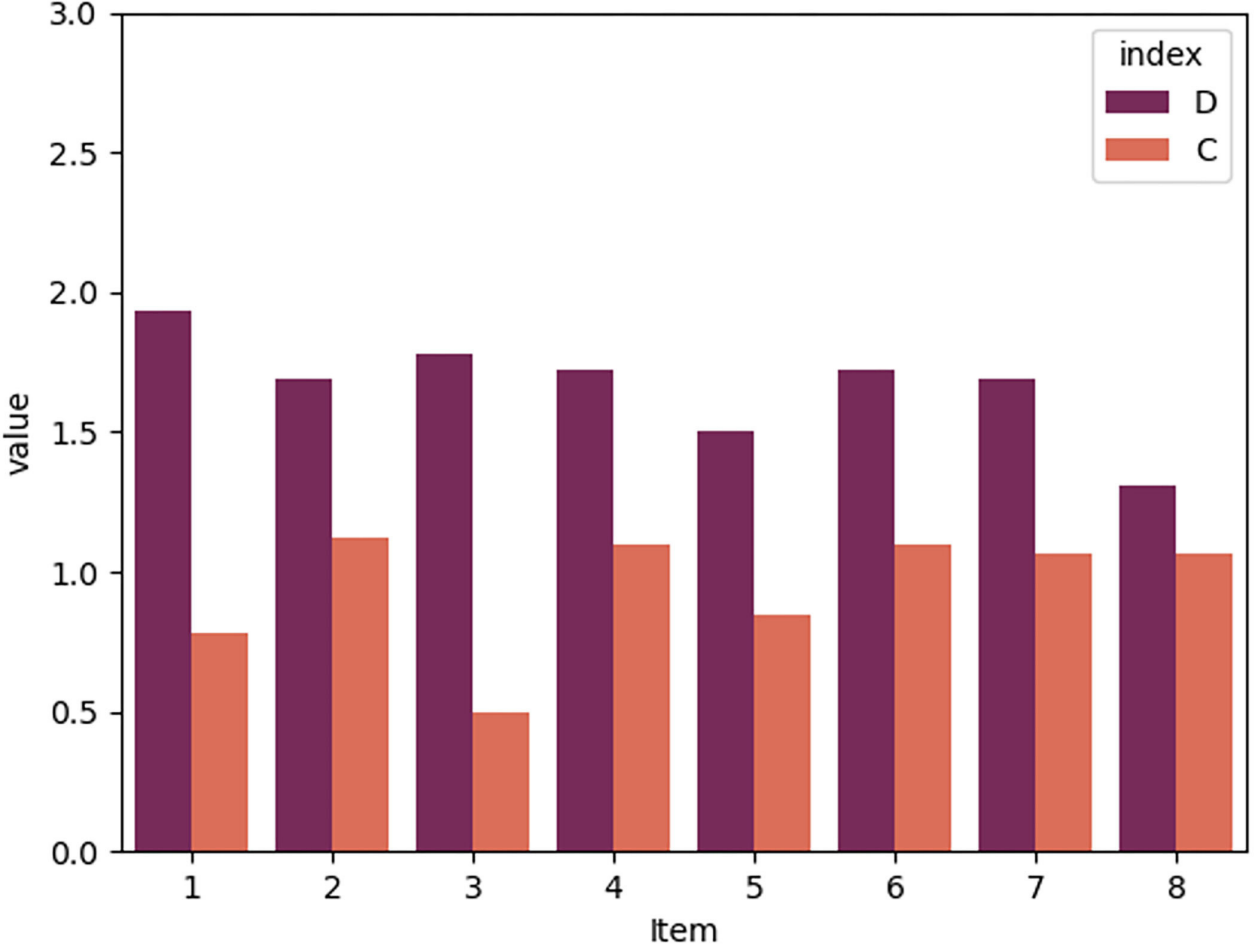
The User Experience Questionnaire (UEQ)-S results. D, dashboard; C, resource comparison. Items: (1) confusing/clear, (2) inefficient/efficient, (3) complicated/easy, (4) obstructive/supportive, (5) boring/exciting, (6) not interesting/interesting, (7) conventional/inventive, and (8) usual/leading edge. The UEQ-S scale starts from−3, and the bottom part of the graph was cut off for visual clarity.

The sample (*N* = 32) included representatives of humanitarian professions (*n* = 15) and technical professions (*n* = 13); the participants who chose the option “other” specified their professions as “biology,” “nursing,” and “art.” Mostly quantitative research was conducted by 14 participants and mostly qualitative by 11 participants. In terms of academic literature, 21 participants dealt with it “rather frequently,” and 10 “only occasionally.” The scores on the UEQ-S items for the dashboard and the resource comparison are presented graphically in Figure 3.

It can be seen that the dashboard received higher scores on all items than the resource comparison. In addition, we compared subgroups of participants (humanitarian *vs*. technical professions, quantitative *vs*. qualitative research, dealing with academic literature frequently *vs*. occasionally). For results, see Supplementary Figures 1–3. All subgroups of participants found the dashboard clearer (item 1), easier (item 3), more supportive (item 4), more exciting (item 5), and more interesting (item 6) than the resource comparison. For participants with technical professions, the dashboard was easier than for those with humanitarian professions. In other items, though, humanitarians gave higher scores to the dashboard than technical professionals. Participants who conducted mostly quantitative research found the dashboard substantially easier, more efficient, and more supportive than those who conducted mostly qualitative research. The latter group, though, perceived it as more exciting and more inventive than the former. Participants who dealt with academic literature frequently found the dashboard substantially easier and more exciting than those who dealt with the literature occasionally. The latter group assessed the dashboard as more interesting than the former.

When asked to assess similar services if implemented in their area of research, the participants found the integration of the dashboard and the resource comparison useful, with a score of 4.25 (SD = 0.95) on the scale from 1 to 5. In terms of specific tasks (see results presented in Table 1), the dashboard was evaluated as more helpful than the resource comparison for getting acquainted with a new topic, and the resource comparison for answering a specific question. Both services were found useful for getting an overview of relevant research.

**Table 1:** Responses to section B items.

**Service/item**	**1**	**2**	**3**	**4**	**5**
Dashboard	3.87 (1.04)	3.76 (1.15)	4.10 (0.75)	3.77 (1.10)	3.72 (1.25)
Resource comparison	3.45(1.23)	4.03 (1.00)	4.13 (0.90)	3.60 (1.22)	3.64 (1.13)

*Items: (1) get acquainted with a new topic; (2) answer a specific question; (3) get an overview of relevant research; (4) explore novel methods of scholarly communication; and (5) make their own research visible for others. Responses were on a Likert scale from 1 (not helpful at all) to 5 (very helpful). Standard deviations are given in brackets*.

In their answers to open questions (the responses were removed from the open-access data due to data protection considerations), the participants stressed that both services could be useful for various tasks. The most frequently addressed topic in the comments was ease or difficulty in the use of both services. In accordance with the responses to the UEQ-S, some participants called the dashboard easy to use, while others stated that both services were not very intuitive. Criticism (the direct link to the papers is not provided but hidden two clicks away) and suggestions (highlighting the matching graph when studies are selected) were also aimed at easier use and more coherent presentation of information.

## Discussion

In this study, we presented the implementation and user evaluation of a scholarly knowledge graph-powered dashboard. We constructed the dashboard as a multi-relational dynamic visualization tool at the intersection of computer science, graphic design, and human-technology interaction. Our aim was to widen the scope of scholarly knowledge graph-powered interfaces and explore possible ways of improving the user experience, which would eventually lead to wider acceptance of scholarly knowledge graphs by research communities. The preliminary results of the user evaluation survey indicated that the dashboard was perceived as more appealing (easy to use, interesting, effective, and exciting) than the baseline interface, the ORKG resource comparison. The ease-of-use was the most prominent theme in participants' answers to the open questions, and we received suggestions aimed at the further amendment of the dashboard in this respect. The participants found the dashboard especially useful for getting acquainted with a new topic, which means that novices in various research areas might benefit from using domain-specific dashboards.

The limitations of the study are determined by the fact that the ORKG dashboard was implemented as an experimental interface for the purpose of the preliminary assessment. In comparison to other studies, such as mapping graphene research (Vargas-Quesada et al., 2017) or Science Citation Knowledge Extractor (Lent et al., 2018), the topic that we chose was rather narrow. The implementation might be also improved in terms of accessibility, as it is currently available solely on desktop versions of Firefox and Chrome. For the user evaluation survey, sampling bias should be taken into account: the sample was not random but rather consisted of volunteers interested in scholarly knowledge graphs or in novel technologies in general. In addition, the sample was not as large as to give us enough statistical power for advanced inferential methods. However, we obtained preliminary results, which might be confirmed by further research on a larger sample, and received valuable feedback from the participants' answers to the open questions. For further research, it will be also interesting to combine the inevitable variability of domain-specific dashboards with the standardization required for the user familiarity (Hu et al., 2017), especially in the case of domain novices (Cole et al., 2002). In the frame of systemic approach (Helkkula et al., 2018) adopted in the ORKG, we consider integrating novelty (the dashboard) and familiarity (the resource comparison).

User-friendly interfaces might play a role in facilitating wider acceptance of scholarly knowledge graphs in the academic community, which is a prerequisite of scholarly communication development in the age of digitalization (Guédon et al., 2019) and research practices appropriate for Open Science (Ignat et al., 2021). The ORKG dashboard, which we created with the aim to increase versatility in the presentation of research results, aids the existing ORKG functionality with the visual modality appreciated by the wider audience. Our findings indicate that scholarly knowledge graph-powered dashboards might be a valuable addition to other graph-based interfaces in various academic domains.

## Data Availability Statement

The authors confirm that the data supporting the findings of this study are available within the article as its Supplementary Materials. Answers to open questions were removed due to data protection considerations.

## Ethics Statement

Ethical review and approval was not required for the study on human participants in accordance with the local legislation and institutional requirements. Written informed consent from the participants was not required to participate in this study in accordance with the national legislation and the institutional requirements.

## Author Contributions

OL developed the dashboard code, conceived the survey, analyzed the data, and wrote the first draft of the manuscript. GK, MS, and SA contributed to the discussion of the idea and to the construction of the survey and invited the survey participants. GK contributed to the choice of the topic for the dashboard. MP integrated the dashboard into the ORKG. All authors contributed to the manuscript revision and approved the submitted version.

## Funding

MP, MS, and SA were partially supported by grants of the European Research Council for the project ScienceGRAPH (Grant agreement ID: 819536) and NFDI4DataScience project (DFG project no. 460234259).

## Conflict of Interest

The authors declare that the research was conducted in the absence of any commercial or financial relationships that could be construed as a potential conflict of interest.

## Publisher's Note

All claims expressed in this article are solely those of the authors and do not necessarily represent those of their affiliated organizations, or those of the publisher, the editors and the reviewers. Any product that may be evaluated in this article, or claim that may be made by its manufacturer, is not guaranteed or endorsed by the publisher.
