# Supplementary figures and images for: A Scholarly Knowledge Graph-Powered Dashboard: Implementation and User Evaluation
Source: Front Res Metr Anal. 2022 Jul 19;7:934930. doi: 10.3389/frma.2022.934930 (PMC9343766; doi:10.3389/frma.2022.934930)

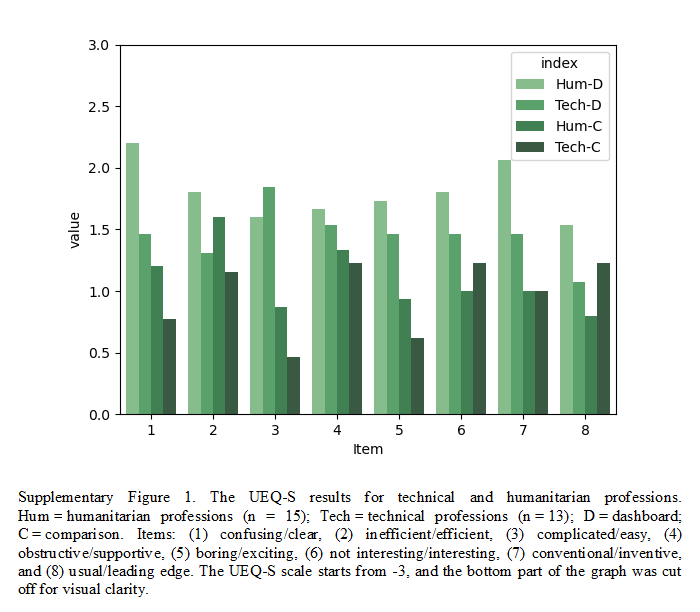

Supplement: Supplementary file 2 [file Image_1.PNG]

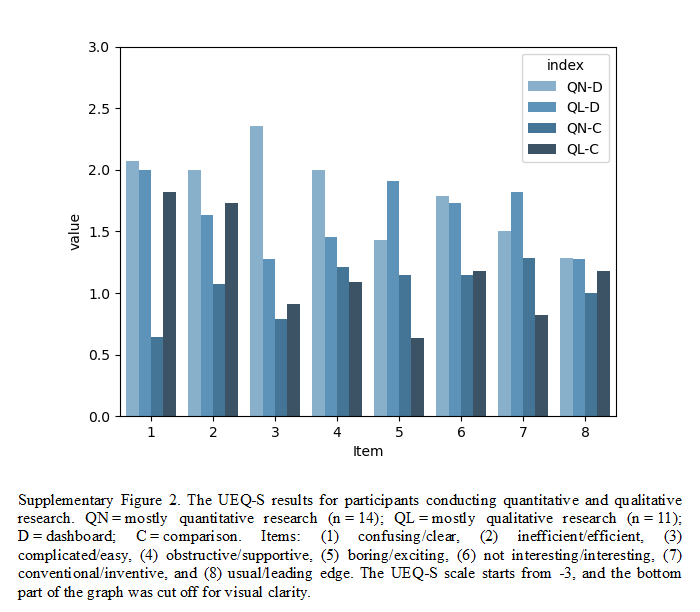

Supplement: Supplementary file 3 [file Image_2.PNG]

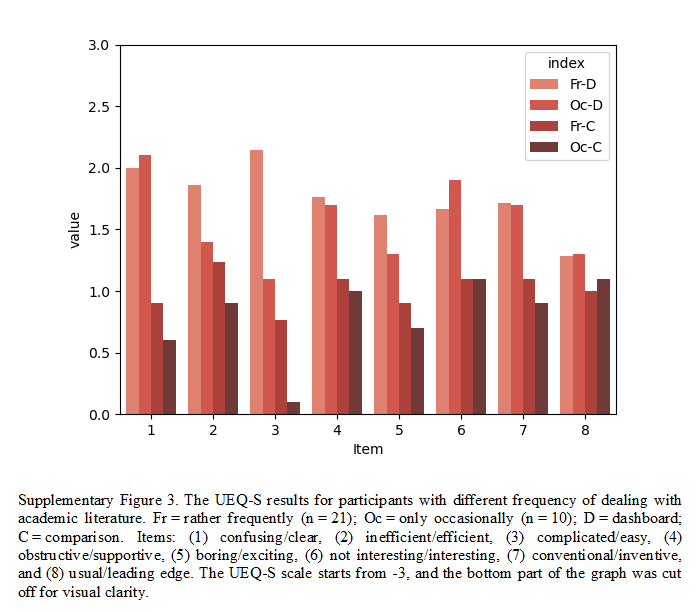

Supplement: Supplementary file 4 [file Image_3.PNG]
